# FAK and paxillin, two potential targets in pancreatic cancer

**DOI:** 10.18632/oncotarget.8040

**Published:** 2016-03-13

**Authors:** Rajani Kanteti, Surinder K. Batra, Frances E. Lennon, Ravi Salgia

**Affiliations:** ^1^ Department of Medicine, Section of Hematology/Oncology, University of Chicago, Chicago, IL, USA; ^2^ Department of Biochemistry and Molecular Biology, University of Nebraska Medical Center, Omaha, NE, USA; ^3^ Department of Medical Oncology and Therapeutics Research, City of Hope, Duarte, CA, USA

**Keywords:** FAK, paxillin, pancreatic cancer, integrins, P53

## Abstract

Pancreatic ductal adenocarcinoma (PDAC) is a devastating cancer in large part due to late diagnosis and a lack of effective screening tests. In spite of recent progress in imaging, surgery and new therapeutic options for pancreatic cancer, the overall five-year survival still remains unacceptably low. Numerous studies have shown that focal adhesion kinase (FAK) is activated in many cancers including PDAC and promotes cancer progression and metastasis. Paxillin, an intracellular adaptor protein that plays a key role in cytoskeletal organization, connects integrins to FAK and plays a key role in assembly and disassembly of focal adhesions. Here, we have reviewed evidence in support of FAK as a potential therapeutic target and summarized related combinatorial therapies.

## INTRODUCTION

Pancreatic ductal adenocarcinoma (PDAC) is a deadly disease and the fourth leading cause of cancer deaths. It has one of the highest mortality rates for solid tumors and the overall five year survival rate is unacceptably low [1]. Invariably, the initial diagnosis occurs at a stage when the cancer is already advanced leading to poor prognosis [2]. Surgery is possible only in 25% of the patients and the gain in survival rises from a dismal 5% to a modest 25% [3, 4]. Tumor cell migration and invasion are the most critical steps in progression of pancreatic cancer and they both occur at an early stage. PDACs are surrounded by dense fibrous tissue due to the intense desmoplastic fibrotic response, one of the hall marks of pancreatic cancer [5]. Some of the associated risk factors PDAC are diabetes, chronic inflammation (pancreatitis), alcohol and cigarette smoking. Tobacco smokers are three times more prone to develop PDAC than nonsmokers[6]. In addition, hepatitis B or C infections can also increase the incidence of PDAC[7]. There are no effective PDAC screening tests available. Adjuvant therapies include fluorouracil-based chemo radiation (fluorouracil and gemcitabine) [8, 9]. However the benefits are marginal as the cancer rapidly acquires drug resistance [10, 11].

In PDAC, the pancreatic stellate cells (PSCs), responsible for the generation of fibrous tissue in the pancreas, play a vital role in tumorigenesis. During development of pancreatic cancer or during inflammation, PSCs undergo morphological and functional changes to become myofibroblast -like cells. They express α-smooth muscle actin (α-SMA) and have high capacity to produce ECM proteins like type-1 collagen and fibronectin. Key intracellular signaling pathways like mitogen activated protein kinases (MAPK), cytokines, growth factors and microRNAs activate PSCs and promote interaction between PSCs and cancer cells that further fuel pancreatic cancer development [12–14]. *In vitro*, PSC culture supernatant stimulated migration, invasion and colony formation of pancreatic cancer cells. In addition, injection of PSCs along with PDAC cells into orthotopic murine models increased tumorigenicity along with metastasis [15, 16]. PSCs are known to accompany cancer cells to metastatic sites and stimulate angiogenesis [17]. Recently Lu J. et al., using a modified Boyden chamber assay, showed that PSCs stimulate migration of pancreatic cells *via* haptotactic mechanisms, which are mediated through collagen-1, activated α2/β1 integrin-FAK signaling pathway [18]. Apart from their indispensable role in fibrogenesis, PSCs through their secretion of matrix metalloproteinases (MMP) and their inhibitors (tissue inhibitors of metalloproteinase, TIMPs) have the potential to promote metastasis [3, 19].

About 10% of the patients inherit PDAC, an aspect that was recently reviewed and will not be addressed here [20, 21]. The inheritance of familial pancreatic cancer (FPC) is mostly autosomal dominant with a heterogeneous phenotype. Germline mutations in BRCA2, PALB2 and ATM are known to trigger pancreatic cancer in some families [22]. Lipocalin-2 and tissue inhibitor of metalloproteinase 1 have recently been identified as potential serum markers for early detection of FPC [23].

**Figure 1:**
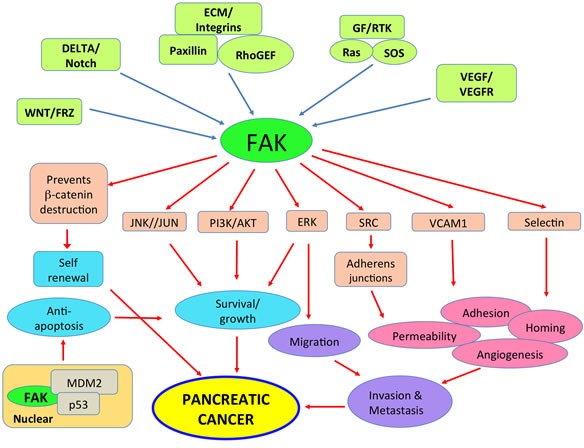
FAK plays a significant role in multiple signaling pathways that contribute to pancreatic cancer growth and metastasis Several receptor systems induce FAK activation that then contributes to the unique function. For instance, RTK signaling through FAK contribute to pancreatic tumor growth and metastasis; however VEGFR mediated signaling through FAK triggers angiogenesis. In addition, K-RAS, which is frequently mutated in pancreatic cancer, is also linked to FAK. FAK also influences lamellipodia formation through activation of small GTPases and promotes homotypic cell adhesion indirectly through paxillin. Suppression of p53 expression by nuclear FAK may also indirectly contribute to tumor growth by inhibiting apoptosis. It is therefore very likely that there is subtle compartmentalization of FAK in the cell and the final effector function could be the result of a combination of FAK mediated and non-FAK mediated signals.

Pancreatic cancer is characterized by several chromosomal abnormalities. There are frequent losses in multiple chromosome arms including 1p, 3p, 4q, 6q, 8p, 9p, 12q, 17p, 18q, and 21q and gains in 8q and 20q [24]. A seminal paper by Kinzler and coworkers [25] described detailed gene expression analysis of tumor transcripts amplified from 24 pancreatic cancers. The transcripts represented more than 23,000 genes. They identified 12 core cellular signaling pathways that favored pancreatic cancer tumor growth and metastasis which were genetically altered in 67-100% of the tumors. Here we highlight, in particular, those pathways involving FAK and paxillin as potential therapeutic targets in pancreatic cancer Figure 1 [26].

## FOCAL ADHESION KINASE (PTK2)

FAK is an intracellular, highly conserved, non-receptor tyrosine kinase encoded by *PTK2* located on human chromosome 8q24.3. It is ubiquitously expressed in all cells [27, 28] and was initially identified in v-Src transformed chicken embryo fibroblasts [29]. FAK is associated with many aspects of metastasis such as adhesion, migration and invasion. FAK is overexpressed and activated in a variety of cancers including colon, breast, lung, thyroid, head and neck, liver, pancreatic and esophageal and is correlated with poor survival rates [30, 31]. The underlying mechanism of FAK overexpression is unclear. FAK is upregulated in PDAC and this increased expression is correlated with the size of the tumor [32].

**Figure 2:**
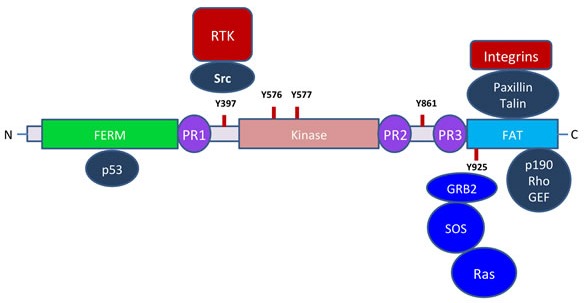
FAK and its potential interacting partners In cancers, highly active receptor tyrosine kinases such as MET and EGFR phosphorylate Src that then interacts with FAK Y397 through SH2 domain. Juxtaposed Src phosphorylates FAK at Y576 and Y577 and possibly other sites, resulting in a highly active FAK both in terms of the kinase activity and as an adapter protein. In addition, it is also connected to RAS through GRB2 and SOS that feeds into MAPK pathway. The CT FAT domain interacts with paxillin that binds to the relatively short cytoplasmic tails of integrins. Although the FERM domain is capable of interacting with integrins and RTKs, the more favored interaction appears to be through paxillin anchored to focal adhesions.

FAK serves as a scaffolding protein and an integral component of focal adhesions and is anchored *via* paxillin. It regulates paxillin function *via* phosphorylation and plays an important role in lamellipodia formation and cell motility. Figure 2 describes in brief, some of the key signaling molecules that FAK interacts with. The 125 kDa FAK protein is mainly composed of N-terminal FERM domain with an autophosphorylation site (Y397), followed by a proline rich region (PR1), central catalytic kinase domain, two additional proline rich regions (PR2 and PR3) and a C-terminal focal adhesion-targeting (FAT) domain (Figure 2). The FERM domain of FAK is structurally similar to cytoskeletal proteins such as talin and the ezrin-radixin-moesin (ERM) family of proteins and also signaling molecules such as the JAK family tyrosine kinases and tyrosine phosphatases [33, 34]. It mediates FAK interaction with integrins and growth factor receptors [27, 35, 36]. The N-terminal PR1 region serves as a docking site for SH3-containing proteins such as cellular Src, whereas the C-terminal PR2 and PR3 regions mediate interactions with other SH3-containing proteins such as p130Cas, endophilin A2, Graf, and ASAP1 [37–39]. The catalytic kinase domain of FAK is highly conserved and contains major phosphorylation sites Y576 and Y577and the ATP binding site K454 [40]. The crystal structure of FAK kinase domain shows an open confirmation, which is very similar to the fibroblast growth factor receptor-1 (FGFR-1) and vascular endothelial growth factor receptor (VEGFR) [41]. The binding of p130Cas with FAK plays an important role in promoting cell migration, which is mediated through RAC activation whereas the binding of FAK with GRAF and ASAP1 regulates cytoskeletal dynamics and focal contact assembly. The FAT domain is mainly required for interactions with other scaffold proteins such as paxillin or talin during focal adhesion formation [42–46]. The C-terminal domain of FAK is known as FRNK (FAK-Related Non-Kinase) acts as a negative regulator of FAK activity by inhibiting activation and signaling of endogenous FAK [47]. FRNK is expressed in significant quantities during neonatal development in vascular smooth muscle cells; however in adults its expression is highly down regulated. Overexpression of FRNK in cells is known to inhibit cell migration; cell spreading and growth factor induced signaling to MAP kinase. Suppression of FAK kinase activity by FRNK obviously can affect both kinase and scaffold mediated FAK functions; however the *in vivo* effects of FRNK in adult remain to be seen [48]. FAK activity is further regulated through its interaction with the FAK-inhibitory protein FIP200. The suppressor of cytokine signaling (SOCS) proteins also interacts with FAK and facilitates the poly-ubiquitination of FAK and its degradation [49].

## ACTIVATION OF FAK BY INTEGRINS

**Figure 3:**
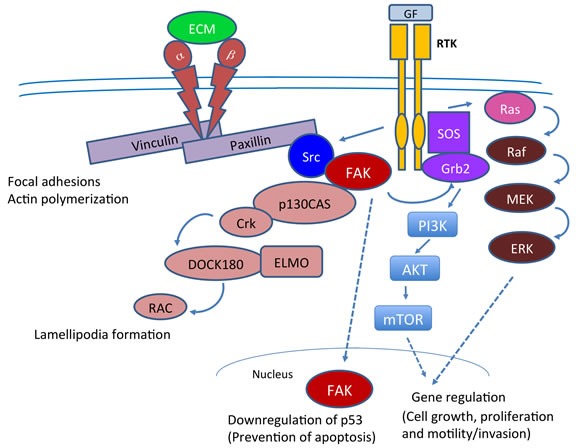
Cell signaling pathways in which FAK plays a key role in tumorigenesis FAK plays a central role in both Integrin and RTK mediated signaling and plays a key role in cytoskeletal changes, lamellipodia formation and cell proliferation and motility. Interaction of integrins with ECM also triggers downstream activation of FAK resulting in its autophosphorylation at Y397. In parallel, the RTK mediated activation of c-Src and its related kinases such as Fyn and Lck, results in the generation of an open SH2 domain that docks on to Y397 and further propagation of the signals. Src mediated Y925 phosphorylation recruits GRB2 which then leads to the activation of RAS and subsequently ERK2. The Y925 site in FAK is a part of paxillin interaction site and its phosphorylation is likely to disrupt FAK localization to focal adhesions. Interestingly, ERK2 mediated FAK phosphorylation at S910 also destabilizes paxillin-FAK interaction. Moreover, ERK2 mediated phosphorylation of paxillin can positively influence FAK adherence to focal adhesions. FAK thus promote cell proliferation and motility that ultimately translates to cancer metastasis. In addition, FAK has the potential to interact with p53 promoter site and down regulate p53 transcription, thus promoting cell survival.

Signaling pathways mediated by integrins play a critical role in cancers in general and pancreatic cancers in particular (Figure 3). Integrins belong to family of transmembrane receptors and they link extracellular matrix proteins (ECM) with the intracellular actin cytoskeleton to regulate cell shape and motility. Integrins are heterodimeric receptors composed of α and β subunits. There are 24 known α subunits and 9 β beta subunits and the particular combination of two subunits (α, β) generate a specific integrin receptor. Each integrin recognizes a specific ligand such as vitronectin, fibronectin, laminin and collagen, which are present in either interstitial spaces or in basement membrane. Integrins also serve as coreceptors to ICAM-1, VCAM-1. Apart from their role in cell adhesion, binding of integrins to ECM proteins activates integrin-associated tyrosine kinases, resulting in the tyrosine phosphorylation of downstream signaling molecules. Figure 3 describes some of the important mediators of these signaling pathways. Thus by controlling these signaling events, integrins play an important role in the regulation of cell migration and cell survival [50, 51]. The relatively short cytoplasmic tails of integrins interact with cytoskeletal adapter proteins, such as paxillin (described below in greater details), which then recruits FAK. Ligand mediated clustering of integrins promotes FAK autophosphorylation at Y397, which then interacts with the SH2 domain of c-Src that is activated by RTKs. This site can also potentially interact with the SH2 containing proteins such as Shc, PI3K, Grb2 and phospholipase C. Binding and subsequent activation of Src leads to further tyrosine phosphorylation of FAK at Y407, Y576 and Y577, in its kinase domain to facilitate maximum activation [35, 52, 53]. Src mediated phosphorylation of FAK at Y925 creates a docking site for GRB2 which activates the small GTP protein RAS and the downstream ERK2 (MAPK) that is known to play an important role in directed cell motility and invasion.

## FAK AND RECEPTOR TYROSINE KINASES

Several of the well-known oncogenes are receptor tyrosine kinases (RTKs) such as EGFR, MET, EPH, VEGFR and KIT. They play an indispensable role in tumor growth and signaling through these receptors also contributes to cancer cell motility and metastasis. This is due in part to their ability to activate FAK and MAPK (Figure 2). The connection between RTKs and FAK appears to be indirect. The most likely scenario is through the ability of RTKs to activate Src family tyrosine kinases (STK) whose SH2 domain binds to FAK. This further feeds signals that promote cell motility.

## FAK AND K-RAS

K-RAS protein is a GTPase, and an early player in the signal transduction pathways regulating cell proliferation and differentiation [54]. Pancreatic cancer has maximum *K-RAS* alterations as compared to any other tumor type. In 75 - 90% of pancreatic tumors, there are activating point mutations of the *K-RAS* oncogene at codons 12, 13 and 61 [55, 56]. The most common of which is substitution of the wild type glycine residue (codon 12) by cysteine, arginine, valine, or aspartic acid [55]. These mutations result in the generation of constitutively active K-RAS. The active K-RAS mutants in turn activate related downstream signaling pathways including RAF-MAPK, MEK1/2 and PI3K-AKT, resulting in uncontrolled cell growth and metastasis.

In pancreatic cancer, *K-RAS* mutations usually develop in the early phase of carcinogenesis and patients with mutated *K-RAS* have significantly less overall survival compared to patients with wild type *K-RAS*. This clearly suggests that mutations in *K-RAS* can initiate and advance pancreatic cancer. Apart from mutations, *K-RAS* is frequently amplified in pancreatic cancer. The role of FAK in K-RAS mediated cell migration and motility is controversial [57, 58]. K-RAS mediated activation of MEK1 results in phosphorylation of FAK at Ser910 resulting in the suppression of its kinase activity [59]. This most likely occurs through activated RAS that signals *via* Fgd1-Cdc42-PAK1- MEK-ERK signaling cascade. MEK1 phosphorylates FAK S910 that results in the recruitment of PIN1 and PTP-PEST. The co-localization with FAK occurs at the lamellipodia of migrating cells. PIN1 binding and prolyl isomerization of FAK can cause PTP-PEST to interact with and dephosphorylate FAK at Y397 resulting in suppression of FAK kinase activity. Despite the inhibition of FAK kinase activity, activated RAS can still promote cell migration, invasion, and metastasis through a FAK independent pathway. It is therefore possible that the use of FAK inhibitors in those pancreatic cancers wherein RAS is highly activated may not be of any use as FAK is already suppressed. This however remains to be seen as FAK is known to be highly active in majority of pancreatic cancers [32]. It is also noteworthy that despite the significant role played by mutated *RAS* in various cancers, to date no successful treatment has been developed that is based on targeting RAS.

## FAK AND PAXILLIN

Paxillin is a major component of focal adhesions that form a structural link between extracellular matrix and actin cytoskeleton. It is a multidomain, 68 kDa protein, first identified as a phosphotyrosine protein in cells transformed by v-Src [60, 61]. In cancer cells, its function is regulated through Src and FAK mediated phosphorylation [62, 63]. Paxillin lacks any intrinsic enzymatic activity and functions mainly as an adaptor protein, by creating an array of docking sites for other proteins, thus promoting the assembly of multiprotein complexes.

Active FAK is located at the leading edge of the cell where it regulates the assembly and disassembly of focal adhesions that result in directional movement. Paxillin appears to be responsible for the disassembly of adhesions at the cell front. This is mediated by the interaction FAK/Src complex with paxillin. A recent study indicates that paxillin recruitment to the cell front protrusions occurs after the assembly of the focal adhesion [64]. Several studies have now shown that both FAK and paxillin are important for cell migration and participate in the dynamic assembly and disassembly of focal adhesions [65–68].

The interaction between paxillin and p130Cas with FAK is through the FAT domain in FAK and appears to be constitutive. The SH3 mediated binding of p130Cas to FAK results in the increased tyrosine phosphorylation of p130Cas at multiple sites. This in turn stimulates SH2 mediated binding of Crk adaptor protein to p130 Cas ultimately resulting in the activation of RAC mediated membrane ruffling or lamellipodia formation and the promotion of cell motility/invasion [69].

The significance of paxillin and FAK interaction can be appreciated by the recent report that a FAK mutant which fails to interact with paxillin resulted in a decrease in FAK in focal adhesions and a noticeable decrease in its phosphorylation. This ultimately led to a significant reduction in adhesion, migration and invasion. It is therefore clear that targeting FAK in cancer therapy is likely to also suppress paxillin-mediated functions [70]. In this fashion, paxillin could serve as a biomarker for FAK therapeutics.

Paxillin also interacts with various other structural proteins such as vinculin and actopaxin, regulators of actin organization (COOL/PIX and PKL/GIT) and the adaptor protein Crk. Through these protein-protein interactions paxillin plays a pivotal role in various physiological processes such as gene expression, matrix organization, tissue remodeling, cell proliferation, cell survival, cell motility and metastasis [44, 46, 71].

Hic-5 and Leupaxin are related to paxillin and are frequently found to coexist. While paxillin is ubiquitously expressed in most of the tissues (except for nervous system) [72–74], Hic-5 is mostly expressed in smooth muscle tissues, particularly the vasculature [75, 76] and Leupaxin is mainly expressed in leukocytes [77]. Gene knockout studies in mice resulted in distinct phenotypes for paxillin and Hic-5. Loss of paxillin resulted in early embryonic lethality whereas the Hic-5 knockouts were viable with minor vascular defects [78, 79] indicating that paxillin interactions are more vital.

The carboxy terminal of paxillin is composed of four LIM domains, which are zinc-binding structures, whereas the amino terminal has five LD motifs and multiple SH2- binding domains. LIM domains are double zinc finger motifs that target paxillin to focal adhesions. LD motifs mainly function as binding sites for other proteins. Moreover, the N terminal region of paxillin has a proline rich region that serves as a dock for SH3 containing proteins. There are several alternatively spliced isoforms of paxillin that, based on their expression levels, could influence the overall function, however this aspect needs further study [72, 80].

Paxillin is known to acquire gain of function mutations that are associated with alterations in the malignant progression of many tumors [81–83] including breast, lung [84–86], prostate [87], melanoma [88] and colorectal cancer [89]. The most common mutation, A128T as identified from our laboratory, is linked to invasive tumor growth [79, 84, 90–92].

In addition to integrin signaling, paxillin also plays a vital role in RTK mediated signaling which is especially important in tumor growth and metastasis. EGF, TGF-β, platelet derived growth factor (PDGF) and androgen receptor mediated signaling are all known to induce paxillin phosphorylation, an event linked to tumor growth and metastasis [93–97]. Paxillin expression levels are known to correlate with HER2 levels in breast cancer cells and patient samples and thus may be a predictor of therapeutic efficacy [83, 98].

Paxillin and its relative Hic-5 appear to play a key role in invadopodia, which are cell protrusions that aid tumor metastasis. They are rich in membrane-bound and soluble matrix metalloproteinases (MMPs) that dissolve the fibrotic and ECM envelope that surrounds the tumor. They have an actin core and several actin-binding and nucleating proteins including paxillin and Hic-5. In addition, paxillin tyrosine phosphorylation is necessary for promoting invadopodia dynamics. Hic-5 also appears to be an essential component of invadopodia formed in cells that have undergone a TGF-α-mediated EMT and forced expression of Hic-5 in epithelial cells can eliminate the need for TGF-α. Paxillin may be dispensed with in cases wherein Hic-5 expression levels are relatively high. A similar role for leupaxin in cancer cell invadopodia has been noted. The Rho GTPases along with the Rho proteins that are anchored through paxillin appear to act in concert and coordinate invadopodia movements.

Cancer cell motility can be described as mesenchymal or amoeboid and the ability of cancer cells to switch between the two is known as plasticity. Paxillin and Hic-5 play a critical role in breast cancer cell morphology and plasticity during invasion. Abrogation of Hic-5 expression promotes an amoeboid phenotype while suppression of paxillin levels results in favoring of a mesenchymal morphology [99].

Paxillin may have an important role in cervical cancer. It was shown to interact with the bovine form of the human papillomavirus (HPV) E6 protein and promote cell transformation [100–102]. Most importantly, paxillin also regulates anchorage independent growth and cell survival through its own tyrosine phosphorylation, which facilitates interaction with p210BCR/ABL, FAK and vinculin [73, 97, 103–105]. In addition, its interaction with the anti-apoptotic protein BCL-2 appears to promote cell survival in the absence of cell adhesion in both cancerous and normal cells. This is partly mediated through FAK signaling [106, 107]. It has been previously shown that Wnt5A, JNK and paxillin are overexpressed in pancreatic cancer and Wnt5A/JNK signaling stimulates cell migration in pancreatic cancer by activating paxillin [108, 109]. Our lab has studied the effect of activating mutations of paxillin on mitochondrial dynamics in lung cancer. Live cell imaging showed that compared to wild type, some mutant clones had enhanced focal adhesion and lamellipodia formation (A127, P233L and P487L). Paxillin mutants exhibited altered association with BCL-2, Dynamin-related Protein-1(DRP-1) and Mitofusion-2 (MFN-2) proteins resulting in dysregulated mitochondrial dynamics. Our results suggest that paxillin mutants through their interactions with BCL-2 and DRP-1 could regulate cisplatin drug resistance in human lung cancer cells [110].

## FAK AND PI3 KINASE

PI3K is a cellular oncogene and an essential intracellular lipid kinase that plays an important role in cell survival. FAK through phosphorylated Y397 is known to directly interact with the SH2 domain of p85, the regulatory subunit of PI3K[111, 112]. Both SH2 and SH3 domains of p85 appear to play a role in the binding of PI3K to FAK [113]. FAK has recently been shown to activate the PI3K pathway to suppress doxorubicin-induced apoptosis[114]. PI3K-AKT pathway is essential for cell survival and shown to be constitutively active in most PDACs. Targeting this pathway with small molecule inhibitors or by knock down strategies results in growth inhibition, both *in vitro* and *in vivo* [115]. In another study, dual targeting of PI3K-AKT2 with RNA interference resulted in significantly increased apoptosis and reduced proliferation and colony formation *in vitro* and *in vivo*. This clearly indicates that simultaneous targeting of key molecules in PDAC is an effective treatment strategy [116, 117].

## FAK AND TUMOR SUPPRESSOR GENES

### TP53

*TP53* is an important tumor suppressor gene. According to cosmic database 49% of pancreatic cancers have *TP53* mutations [118]. MK 1775 a Wee 1 inhibitor targets aberrant p53 by blocking cell cycle checkpoint regulation [119]. The interaction between p53 and FAK appears to serve two purposes. The fact that FAK has been discovered in nucleus and that it can bind to p53 and inhibit apoptosis supports a role for FAK activity in tumorigenesis. It was also found that p53 can bind to the FAK promoter site in the nucleus and suppress its transcription thereby aiding p53-mediated cell cycle arrest and apoptosis [120].

### SMAD4

In normal cells, the cytokine TGF-β acts, as a tumor suppressor; however in cancer cells it is known to promote metastasis. It signals through SMAD4, a transcription cofactor, that stimulates gene transcription. SMAD4 is known interact with FAK. There appears to be a cross talk between TGF-β and RTKs. TGF- signaling is also known to induce clustering of some RTKs through FAK. Moreover 20% of pancreatic cancers are known to harbor *SMAD4* mutations that correlate with poorer prognosis and increased metastasis [121, 122].

### MERLIN

Merlin is a tumor suppressor protein, coded by the *NF2* gene (Neurofibromatosis type II). It is a membrane cytoskeleton protein, which links actin filaments to the cell membrane, and mediates tumor suppression through contact-mediated growth inhibition [123, 124]. It has been shown that *NF2* function or expression is lost in various cancers through mutation or chromosome deletion [125–127]. This is particularly relevant in malignant mesothelioma where 50% of patients have loss or inactivation of Merlin. Merlin deficient cancer cells, including mesothelioma cells are very sensitive to FAK inhibition. A study by Shapiro and coworkers reported that mesothelioma cells showed increased sensitivity to the small molecule FAK inhibitor VS-4718 *in vitro* as well as *in vivo* tumor xenograft models. This study clearly demonstrated a synthetic lethal relationship between Merlin loss and FAK inhibitor sensitivity in MPM [128].

## FAK INHIBITORS

Several approaches are used to target and inhibit FAK activity in cancer cells. Initial trials included, knockdown of FAK using siRNA, antisense oligonucleotides and adenoviral dominant-negative FAK-CD. These methods induced significant FAK down regulation, inhibited cancer cell proliferation, increased apoptosis and thus decreased tumorigenicity [129–131]. However these approaches have limitations for clinical research because of their toxicity *in vivo*. This led to development of small molecule inhibitors of FAK. These are divided mainly into two groups. The first group is comprised of inhibitors that target enzymatic or catalytic kinase dependent functions of FAK. The other group consists of the compounds that inhibit kinase independent functions of FAK, such as its protein-protein interactions with other binding partners [53, 132, 133].

### FAK Kinase inhibitors

These are ATP analogs that effectively suppress the kinase activity of FAK [134–137]. These inhibitors bind to the residues surrounding ATP- binding pocket of kinases and since this pocket is similar in majority of the kinases, they unfortunately tend to have far off target effects. The most well-known and specific FAK inhibitors are either pyrimidine (NVP-TAE-226, PF-573228, PF-562271 and GSK2256098) or pyridine based (VS-6063, VS-4718 and VS-5095).

### FAK Scaffold inhibitors

FAK is known to interact with many proteins such as Paxillin, Src, EGFR, Her-2, MET, PI3K, VEGFR-3 and the scaffolding function of FAK plays an important role in cancer cell signaling. Disrupting the formation of these complexes can inhibit downstream FAK signaling in cancer cells.

All the recent inhibitors are listed in the Table 1.

**Table 1:** Inhibitors of FAK kinase and scaffold function

Name	Target	Specificity	Cancers targeted	Clinical Trial	References
TAE-226 Novartis	Kinase inhibitor ATP competitive	FAK & PYK2	Glioma & ovarian	Preclinical	136, 143,144
PF-573,228 Pfizer	Kinase inhibitor ATP competitive	FAK	Prostate & breast	Preclinical	137
GSK2256098 GlaxoSmithKline	Kinase inhibitor ATP competitive	FAK	Ovarian & pancreatic	Phase I	26
NVP-TAC544	Kinase inhibitor ATP competitive	FAK	N/A	Preclinical	26
VS-4718 (PND-1186) Verastem	Kinase inhibitor ATP competitive	FAK & PYK2	Breast & ovarian	Phase I	26
VS-6062 (PF562271 and PF271) Verastem	Kinase inhibitor ATP competitive	FAK & PYK2	Breast, prostate, pancreatic, head & neck	Phase I	26, 135
VS-6063 Verastem	Kinase inhibitor ATP competitive	N/A	Ovarian	I/Ib and II	26, 151
1H-Pyrrolo(2,3-b) Merk Serono	Kinase inhibitor Non-ATP competitive	Hinge region of FAK	N/A	Preclinical	138
Compound 1 and 2 Takeda	Kinase inhibitor Non-ATP competitive	FAK Y397 site	N/A	Preclinical	138
Y15 (Compound 14) Cure FAKtor Pharmaceuticals	Kinase inhibitor Non-ATP competitive	FAK Y397 site	Colon	Preclinical	133, 146, 147
C4 Cure FAKtor Pharmaceuticals	Scaffold inhibitor	FAK /VEGFR pathway	Pancreatic & breast	Preclinical	148
R2 (Roslins) Cure FAKtor Pharmaceuticals	Scaffold inhibitor	FAK & p53	Colon	Preclinical	149
Y11 Cure FAKtor Pharmaceuticals	Scaffold inhibitor	FAK Y397 site	Colon & Breast	Preclinical	26

### FAK and combinatorial therapy

Multiple signaling pathways such as those mediated through RTKs, FAK, Src, AKT, MAPK and PI3K/mTOR are known to play important roles in tumorigenesis and also contribute to drug resistance. A strategy specifically to overcome resistance of cancer cells to chemotherapy and also to increase the efficacy of drugs is to use a combinatorial approach. A combination of FAK inhibitors along with inhibitors of other signaling molecules has proven more effective than single drug alone [138].

The dual inhibition of FAK with a dominant negative form FAK-CD and EGFR inhibitor AG-1478 or Src inhibitor PP2 was much more effective as compared to FAK inhibition alone. This combination treatment resulted in increased cell detachment, inhibition of AKT/ERK1/2 and Src, and increased apoptosis, as evidenced by increased cleaved caspase 3 and 8 in breast and colon cancer cells [139, 140].

TRAIL is known to induce apoptosis in cancer cells; however its clinical use has been limited due to rapid development of resistance [141]. Dao et al., using PANC-1 cells (TRAIL resistant pancreatic cancer cells), showed that PH11, a novel Focal Adhesion Kinase (FAK) inhibitor in combination with TRAIL rapidly induced apoptosis. PH11 appeared to downregulate c-FLIP *via* inhibition of FAK and the PI3K/AKT pathways, thereby rendering PANC-1 cells susceptible to TRAIL induced apoptosis [142].

The combination of FAK inhibitor TAE-226 and docetaxel, an antimitotic drug, demonstrated a significant decrease in ovarian tumor growth, increased apoptosis in endothelial cells, reduced microvessel density and prolonged survival [143]. In another study it was shown that TAE-226 also increased radio-sensitivity of head and neck cancer cells [144]. The combination of FAK inhibitor PF-562271 with SU11248, an angiogenesis inhibitor, decreased tumor growth and inhibited angiogenesis of human hepatocellular carcinoma in rat xenograft model [145]. The combination of FAK inhibitor Y15 with Src inhibitor PP2 significantly decreased viability of colon cancer cells along with decrease in Y397 FAK and Y418 Src phosphorylation [146]. Also recently it was shown that the combination of FAK inhibitor Y15 with gemcitabine was more effective in suppressing tumor growth in a pancreatic cancer mouse xenograft model than the use of a single drug alone [147]. The combination of another FAK inhibitor, C4 with doxorubicin was more effective in inhibiting breast cancer xenograft tumor growth and angiogenesis in mice [148]. In another study Y15 was used in combination with temozolomide and was shown to be very effective in blocking U87 glioblastoma xenograft tumor growth model [149]. Roslin (R2), a small molecule that disrupts the interaction of FAK with P53 significantly decreased tumor growth in colon cancer. It also sensitized HCT16 cells to doxorubicin and 5-fluorocil [150].

A recent development in cancer therapeutics further strengthens FAK as a viable cancer therapeutic target. In ovarian cancers resistant to taxane treatment, it was observed using reverse-protein arrays, that levels of YB-1, a RNA binding protein, which regulates transcription, are elevated. AKT mediated phosphorylation of YB-1 promotes resistance; however FAK inhibition prevents this, thereby making these cancers susceptible to taxane treatment [151]. The above studies further reinforce the importance of combination therapy, where FAK inhibitor sensitizes cancer cells to chemotherapy.

## CONCLUSIONS AND FUTURE PERSPECTIVES

PDAC is a devastating disease with poor prognosis. Of the known signaling pathways that contribute to PDAC, FAK plays a vital role in signaling pathways mediated through integrins, RTKs, RAS, and TGF β Moreover, it is also likely to suppress p53 expression. We therefore speculate that therapeutic targeting of FAK in PDAC is therefore likely to succeed, particularly when this strategy is combined with other chemotherapeutic treatments. Signaling pathways downstream of activated FAK including paxillin will be important to study in the context of FAK inhibition and other therapeutics to identify novel biomarkers. In the future, emerging technologies may allow for direct therapeutic targeting of paxillin.
